# An examination of five theoretical foundations associated with localized thermosensory testing

**DOI:** 10.1007/s00421-021-04670-z

**Published:** 2021-03-25

**Authors:** Mevra Temel, Andrew A. Johnson, George Havenith, Josh T. Arnold, Anna M. West, Alex B. Lloyd

**Affiliations:** 1 School of Design and Creative Arts, Loughborough University, Loughborough, UK; 2 Environmental Ergonomics Research Centre, Loughborough University, Loughborough, UK

**Keywords:** Thermal sensation, Thermal discomfort, Thermosensory, Confidence, Experimental design

## Abstract

**Purpose:**

To assess five theoretical foundations underlying thermosensory testing using local thermal stimuli.

**Methods:**

Thermal sensation, discomfort and the confidence of thermal sensation scores were measured in 9 female and 8 male volunteers in response to 17 physical contact temperature stimuli, ranging between 18–42 °C. These were applied to their dorsal forearm and lateral torso, across two sessions.

**Results:**

Thermal sensation to physical temperature relationships followed a positive linear and sigmoidal fit at both forearm (*r*^*2*^ = 0.91/*r*^*2*^ = 0.91, respectively) and lateral torso (*r*^*2*^ = 0.90/ *r*^*2*^ = 0.91, respectively). Thermal discomfort to physical temperature relationships followed second and third-order fits at both forearm (*r*^*2*^ = 0.33/*r*^*2*^ = 0.34, respectively) and lateral torso (*r*^*2*^ = 0.38/*r*^*2*^ = 0.39, respectively) test sites. There were no sex-related or regional site differences in thermal sensation and discomfort across a wide range of physical contact temperatures. The median confidence of an individual’s thermal sensation rating was measured at 86%.

**Conclusion:**

The relation between thermal sensation and physical contact temperature was well described by both linear and sigmoidal models, i.e., the distance between the thermal sensation anchors is close to equal in terms of physical temperatures changes for the range studied. Participants rated similar thermal discomfort level in both cold and hot thermal stimuli for a given increase or decrease in physical contact temperature or thermal sensation. The confidence of thermal sensation rating did not depend on physical contact temperature.

## Introduction

Research into local thermal, mechanical, and wetness sensation has provided a wealth of knowledge that has successfully developed our basic understanding of the neurophysiological responses to external temperature stimuli (Havenith et al. 1992; Stevens and Choo 1998; Fukazawa and Havenith 2009; Ouzzahra et al. 2012; Filingeri et al. 2013, 2014b, 2015; Gerrett et al. 2015a). In addition to this, a large body of literature has investigated subjective thermal sensations evoked in response to the application of conductive cold and hot stimuli to localized areas of skin (Stevens et al. 1974; Nakamura et al. 2008; Ouzzahra et al. 2012; Gerrett et al. 2014, 2015b; Coull 2019). This knowledge has been successfully applied to the domains of clothing design, comfort assessment, and sport sciences (Fukazawa and Havenith 2009; Ouzzahra et al. 2012; Gerrett et al. 2013; Raccuglia et al. 2016, 2017, 2018a, b).

Recent studies have, however, queried some of the principles and assumptions underlying our interpretation of thermal subjective responses to temperature and wetness stimuli. For example, Raccuglia et al. (2018a) demonstrated that thermosensory responses over time are prone to influence from a previous rating (i.e., anchoring bias), which can result in systematically higher sensation scores than those truly experienced. Likewise, it has been suggested that the sensory magnitude between verbal anchors on thermal sensation and comfort scales are not perceived as equal; while the relationship between thermal sensation and thermal comfort is largely variable across individuals, and interdependent on contextual factors, such as the residing climate, ethnicity or native language (Havenith et al. 2020; Schweiker et al. 2020).

While research has begun to identify some of the key factors impacting subjective ratings of the thermal environment, a range of theoretical foundations underpinning thermosensory testing remain to be investigated, especially in relation to the localized application of thermal, mechanical, and wetness stimuli. This study was therefore designed to provide empirical evidence and examine the following five theoretical foundations.

### Theoretical foundation 1: thermal sensation relates linearly to physical contact temperature across a wide range

Recent research has widely adopted the application of a conductive thermal probe to the skin of human participants to investigate inter-individual and regional variability in the response to hot, cold, and wet thermosensory stimuli (Stevens et al. 1974; Nakamura et al. 2008; Ouzzahra et al. 2012; Filingeri et al. 2014a; Gerrett et al. 2015a; Coull 2019). Previous research has demonstrated a linear positive relationship between thermal sensation and air temperature during whole-body exposure (Chatonnet and Cabanac 1965; Gagge et al. 1967; Zhang et al. 2004; Wang et al. 2015), however, the assumption that thermal sensations evoked during the application of a conductive thermal probe would also be linearly related to the stimulus temperature, may not be well founded, and has not been researched. Alternative theoretical foundations could include for example a sigmoidal relationship, in which any given increment in physical temperature would elicit a greater change in thermal sensation when close to the thermoneutral zone, compared with the same physical temperature increment applied at the extremes of hot and cold. Therefore, the present study was designed to investigate whether the relationship between a large range of physical contact temperatures and thermal sensations is truly linear between two fixed limits of 18–42 °C, as has been reported during whole-body exposure to air.

### Theoretical foundation 2: local thermal discomfort is more sensitive to cold than hot

It is known that sensation and discomfort are distinct phenomena; on one hand, the thermal sensation is the perception experienced by an individual in response to a given physical temperature stimulus (Hensel 1974; Schepers and Ringkamp 2010), while thermal discomfort is expressed as a lack of thermal pleasantness, thermal pleasure, or hedonic satiation (Cabanac 1971; Cabanac et al. 1972; Marks and Gonzalez 1974; Nagashima et al. 2018). Previous research has reported a skewed U-shaped (quadratic or cubic) relationship between thermal discomfort and physical applied temperature, in which participants experience whole-body cold physical temperatures as more uncomfortable than whole-body hot physical temperature (Gagge et al. 1967). Like theoretical foundation 1, this may not be the case for localized discomfort responses to conductive thermal stimuli. Therefore, the second theoretical assumption investigated in this study was that discomfort evoked during the application of conductive thermal probe nonlinearly follows a skewed (towards cold) U-shaped (quadratic or cubic) relationship.

### Theoretical foundation 3: sex differences exist in thermal sensation and discomfort across a wide range of contact temperatures

Prior research on biological sex differences in thermal sensation has found that females are more sensitive in response to fixed hot stimuli in terms of magnitude estimation (Gerrett et al. 2014) and threshold detection (Lautenbacher and Strian 1991), but Stevens and Choo (1998) found no significant difference in sex-related thermal threshold detection to cooling and warming. However, whether there are sex-related, or other interindividual differences, in thermal sensation responses to a wide range of physical contact temperatures is uncertain. The third theoretical assumption investigated in this study was therefore that sex-related differences in thermal sensation and discomfort occur across a wide range of physical contact temperatures (18–42 °C).

### Theoretical foundation 4: regional thermal sensitivity differences exist independently of thermal discomfort changes, across a wide range of contact temperatures

To better characterize human thermal sensitivity to hot and cold stimuli across the body, several studies have noted differences in regional threshold detection and thermal sensations in response to hot and cold contact temperatures (Stevens et al. 1974; Stevens and Choo 1998; Ouzzahra et al. 2012; Gerrett et al. 2014, 2015a). While it is assumed that such changes occur as direct result of temperature sensitivity per se (i.e., differences in the location, density, and distribution of cutaneous thermoreceptors), and/or anatomical differences such as thickness or conductivity between glabrous and non-glabrous skin, previous research has not ruled out a contribution of potential changes in regional discomfort to the observed regional difference in thermal sensation. It is plausible that higher discomfort in a region would elicit a higher thermal sensation, especially in circumstances where no discomfort ratings are requested from the reporting individual. Therefore, the fourth theoretical assumption investigated in this study was whether regional differences in thermal sensation are truly explained by sensitivity per se, or whether in part, these may be confounded by a corresponding change in thermal discomfort.

### Theoretical foundation 5: good levels of confidence exist in thermal sensation ratings across a wide range of contact temperatures

To our knowledge, no previous literature has provided participants with an option to demonstrate the level of certainty/confidence associated with their thermal sensation, comfort, or wetness ratings, and what specific features might affect their confidence in providing an accurate rating. Thus, it is implicitly assumed that the ratings provided in response to such stimuli are assumed to be 100% certain and thereby a precise measurement of an individual sensorial response to that stimulus. The fifth theoretical assumption investigated in this study was whether thermal sensation ratings are provided with close to 100% certainty across the full range of physical contact temperatures.

## Methods

### Overview of the study

**Fig. 1:**
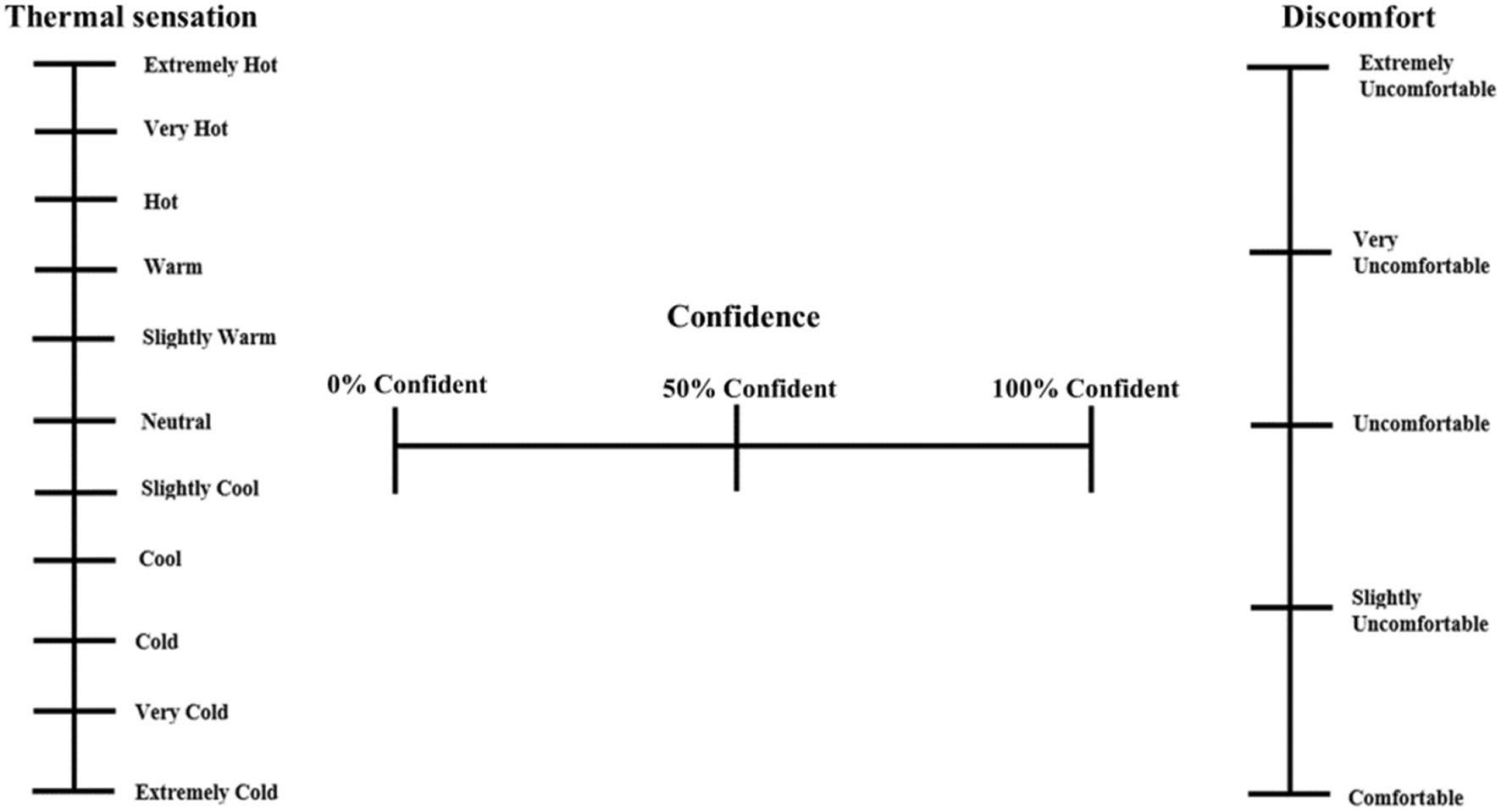
Thermal sensation (left), sensation confidence (middle) and thermal discomfort (right) scales. Extremely cold is 0-mm and extremely hot is 200-mm on the visual analogue scale. 0% confident is 0-mm and 100% confident is 200-mm on the visual analogue scale. Comfortable is 0-mm and extremely uncomfortable is 200-mm on the visual analogue scale. Distances between anchors are equal

To test the theoretical foundations outlined in the introduction of this study, a protocol was developed in which the perceptual responses (thermal sensation, rating confidence, and thermal discomfort; Fig. 1) to the application of 17 absolute physical temperatures, ranging from cold to hot (18–42 °C), were measured. In the first experimental session, the 17 physical temperature stimuli were applied in a mixed counter-balanced order to the left dorsal forearm (a point halfway distal between the antecubital fossa and carpus) of a participant. During a second session, the same 17 stimuli were applied the left lateral torso (at a point that intersects between (a) 10 cm lateral of the midsternal line and (b) 5 cm below of the inframammary line) in a mixed counter-balanced order. Two sites were chosen, one which is highly sensitive to temperature changes (the torso), and one which represents a lower, peripheral temperature sensitivity (the forearm) (Ouzzahra et al. 2012; Gerrett et al. 2014). This protocol characterized the thermal sensation and thermal discomfort relationships to physical temperature in two areas of the body across different sexes, thereby addressing theoretical foundations 1, 2, 3, and 4. To investigate theoretical foundation 5, the present study examined participants’ confidence in their thermal sensation ratings across the range of applied thermal stimuli.

### Participants

Nine female (24.0 ± 4.1 yrs., 63.8 ± 5.3 kg, 167 ± 5 cm, 26.1 ± 3.6% body fat) and eight male university students (23.9 ± 5.0 yrs., 76.4 ± 7.8 kg, 178.8 ± 4.8 cm, 17.2 ± 4.3% body fat) of Western European origin volunteered to participate. Inclusion criteria included: non-smoker, no cardiovascular, musculoskeletal, cutaneous, and metabolic diseases, and no sensory-related disorders. Participants were asked to refrain from the consumption of caffeine or alcohol for a period of 24 hours prior to each trial, and to refrain from the consumption of food for 2 hours prior to each session. The study design was approved by the Loughborough University Ethics Committee (ECC/AJ1) and was conducted within the confines of the World Medical Association Declaration of Helsinki for medical research using human participants.

### Experimental procedures

On arrival to the laboratory, participants were briefed, allowing adequate time to read through the participant information sheet. Written informed consent was then obtained in addition to the completion of a health screening questionnaire. During the experimental trials female participants were asked to wear a sports bra and shorts, while male participants wore shorts. Pre-test measurements included stature, body mass (Kcc150, Mettler Toledo, Leicester, UK), and body fat percentage (BC-418MA, Tanita Corporation, Tokyo, Japan). Following anthropometric measurements, participants entered the environmental chamber (custom designed by TIS Services, UK) regulated at 25 °C and 50% RH. Mean skin temperature was assessed using calculations as proposed by Ramanathan (1964), via wireless temperature loggers (I-Buttons, Maxim, San Jose, USA), appended at four skin sites (left upper chest, left triceps, right anterior thigh and right calf). For the remainder of the trial, participants rested supine (torso risen) on a medical bed. The site of probe application was then marked, ensuring consistent application across temperatures, and all participants were blinded to the environment condition and thermal probe controller unit to prevent any ratings being affected.

A 10-min baseline period was used to familiarize participants with all experimental equipment and subjective scales (Fig. 1). The 11–anchor thermal sensation scale was an extended ASHRAE (7-anchor) scale, adding extremely cold and extremely hot in relation to guidance outlined by the International Standards Organization (NSAI 1998). A three-anchor visual analogue scale was used to measure participant confidence in their thermal sensation response, and a five-anchor visual scale was used for thermal discomfort (regional and whole-body) ranging from ‘Extremely Uncomfortable’ to ‘Comfortable’ (Griffiths and Boyoe 1971; Raccuglia et al. 2016). Each scale was designed so that participants could provide their exact rating on the scale by placing a mark on the scale (i.e., between descriptor anchors), as seen in Fig. 1. Subjects were also informed about a reference temperature equating to extremely cold, such as applying an icepack to their feet, and extremely hot, such as entering a very hot bath. All physical temperatures were applied using a mixed counterbalanced order with a thermal probe (Physitemp Instruments Inc., USA) consisting of a 25 cm^2^ metal surface and with a pressure of 4 kPa. The 17 applied contact temperatures are outlined in Table 1.

**Table 1:** Applied physical temperature stimuli

18 °C	19 °C	20 °C	22 °C	24 °C	25 °C	26 °C	29 °C	30 °C
32 °C	33 °C	36 °C	37 °C	39 °C	40 °C	41 °C	42 °C	–

For all applications, the probe was applied to the skin for a period of 10 s, at the end of which participants rated their thermal sensation, their confidence of thermal sensation, and thermal discomfort. A recovery period of at least 20 s was utilized between thermal probe applications. Prior to each application of the thermal probe, local skin temperature was confirmed to have returned to its baseline value using a single spot infrared thermometer (FLUKE 566, Fluke Corporation, USA). Additionally, during the study, the internal body temperature of each participant was taken at 10 min internals using an aural thermometer (Braun Thermo Scan® PRO 6000, Helen of Troy, USA) and whole-body discomfort was recorded.

### Statistical analysis

Statistical analysis was undertaken using SPSS version 24.0 (IBM, USA). Data are presented as means ± SD. Normality of all data distributions were confirmed using the Kolmogorov–Smirnov test, following which parametric statistics were used. Independent sample *t* tests were conducted on individual characteristics (age, mass, height, and BF) to investigate the difference between two independent groups (female and male). Forearm and torso sites were tested in separate sessions, across different days, and followed the same temperature list in both sessions. Therefore, paired-sample *t* tests were performed on the mean skin temperature and aural temperature to assess the difference between the first session and the second session, and between the start and the end of each session.

Linear and sigmoidal (four-parameter logistic; 4-PL) regression analyses were performed to investigate the relationship between physical contact temperatures and thermal sensation ratings, addressing theoretical foundation 1. Second (quadratic) and third (cubic) order polynomial regression analysis was conducted to analyze the relationship between physical contact temperatures and local discomfort, addressing theoretical foundation 2. To investigate sex and regional differences in the thermal sensation and discomfort to physical temperature relationships (theoretical foundation 3 and 4), a three-way mixed-measure ANOVA analysis (temperature x region x sex; 20 × 2 × 2) was undertaken to examine the main effect of body region and sex on thermal sensation and discomfort at all applied temperatures. Finally, Pearson’s correlation coefficient analysis was conducted to investigate the relationship between the applied physical contact temperatures and confidence of thermal sensation rating, addressing theoretical foundation 5. In all analyses, *p* < 0.05 was used to establish significant differences.

## Results

### Participant characteristics

Male and female participants did not significantly differ in age (*p* = 0.956, 23.9 ± 4.9 vs 24 ± 4.2 yrs.), while males were taller and heavier than females (stature, 178.8 ± 4.8 vs. 167.9 ± 5.1 cm, *p* < 0.001; body mass, 76.38 ± 7.7 vs. 63.8 ± 5.27 kg, *p* < 0.001), and females had higher body fat percentage than males (26.16 ± 3.7% vs 17.2 ± 4.3%, *p*< 0.001).

### Mean skin temperature and aural body temperature

Mean skin temperature did not change between the start and the end of the experiment (for forearm, 32.78 ± 0.3 vs 33.0 ± 0.2 °C, *p* = 0.441; for torso, 32.5 ± 0.2 vs 32.6 ± 0.1 °C, *p* = 0.332, respectively), nor between sessions (forearm, 32.9 ± 0.6 vs torso, 32.9 ± 0.7 °C, *p* = 0.587). Aural body temperature did not change between the start and end of the experiment (for forearm, 36.6 ± 0.3 vs 36.6 ± 0.2 °C, *p* = 0.878; for torso, 36.7 ± 0.2 vs 36.9 ± 0.7 °C, *p* = 0.452, respectively), nor between sessions (forearm, 36.6 ± 0.3, vs torso, 36.7 ± 0.2 °C, *p* = 0.462, respectively). Participants rated their whole-body thermal discomfort level as comfortable during the full duration of the first and second sessions.

### Theoretical foundation 1: thermal sensation

**Fig. 2:**
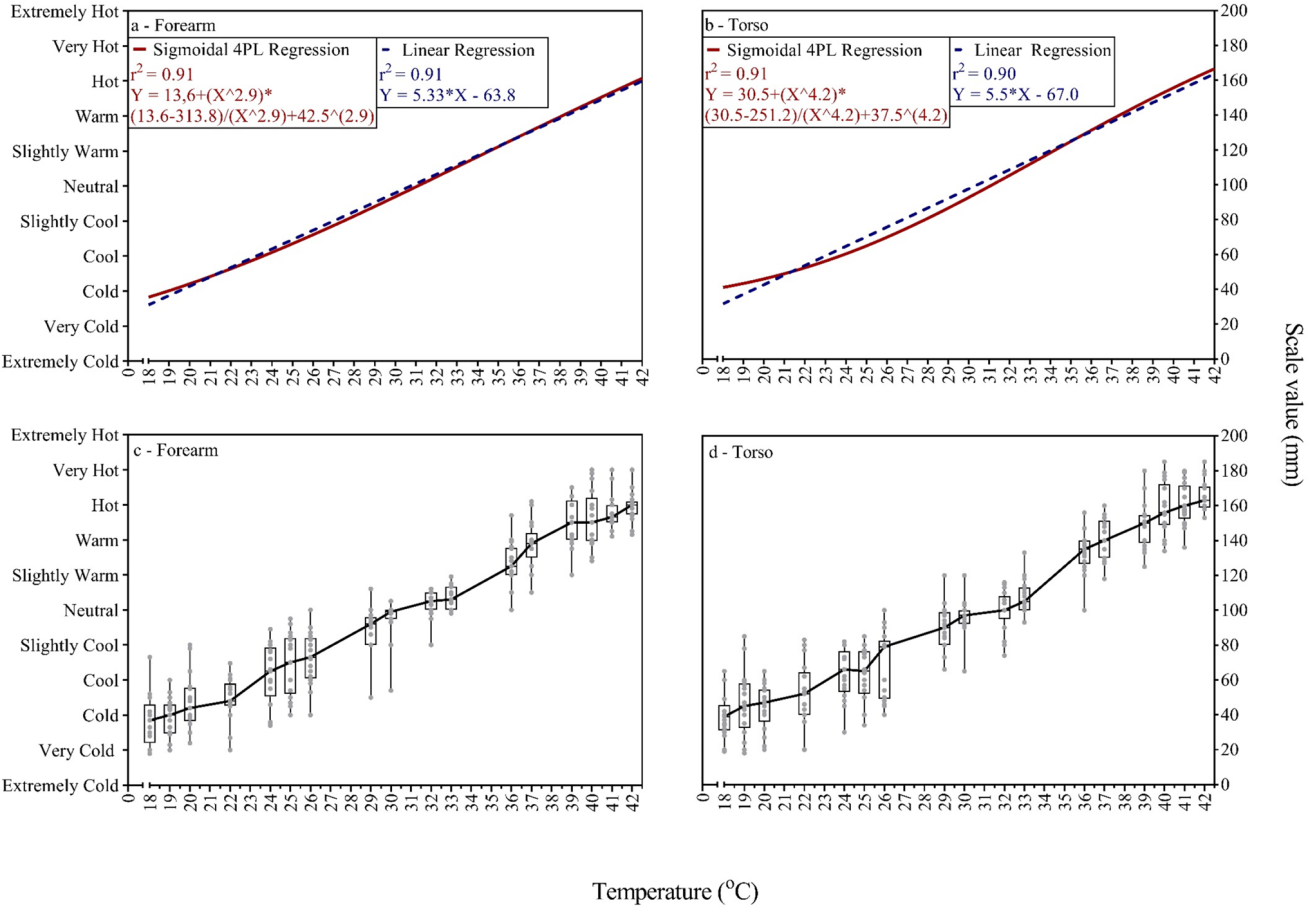
Relationship between applied physical temperature and thermal sensation. Panel **a** and **b** demonstrate linear (blue) and non-linear (4LP-sigmodial) (red) regression lines of thermal sensation in forearm (**a**) and torso (**b**). Panel **c** and **d** demonstrate individual data and box and whiskers with median connection line of thermal sensation in forearm (**c**) and torso (**d**)

Both linear and sigmoidal 4PL regression (Fig. 2, Panel (a–b)) showed similar positive fits at both forearm (*r*^*2*^ = 0.91, *r*^*2*^ = 0.91, respectively and *p* < 0.001 for both) and lateral torso (*r*^*2*^ = 0.90, *r*^*2*^ = 0.91, respectively and *p* < 0.001 for both) test sites. When a cold stimulus of 18 °C was applied on either the forearm or torso, it was perceived between “very cold” and “cool”, while a relatively hot stimulus of 42 °C was perceived between “very hot” and “warm”.

### Theoretical foundation 2: thermal discomfort

**Fig. 3:**
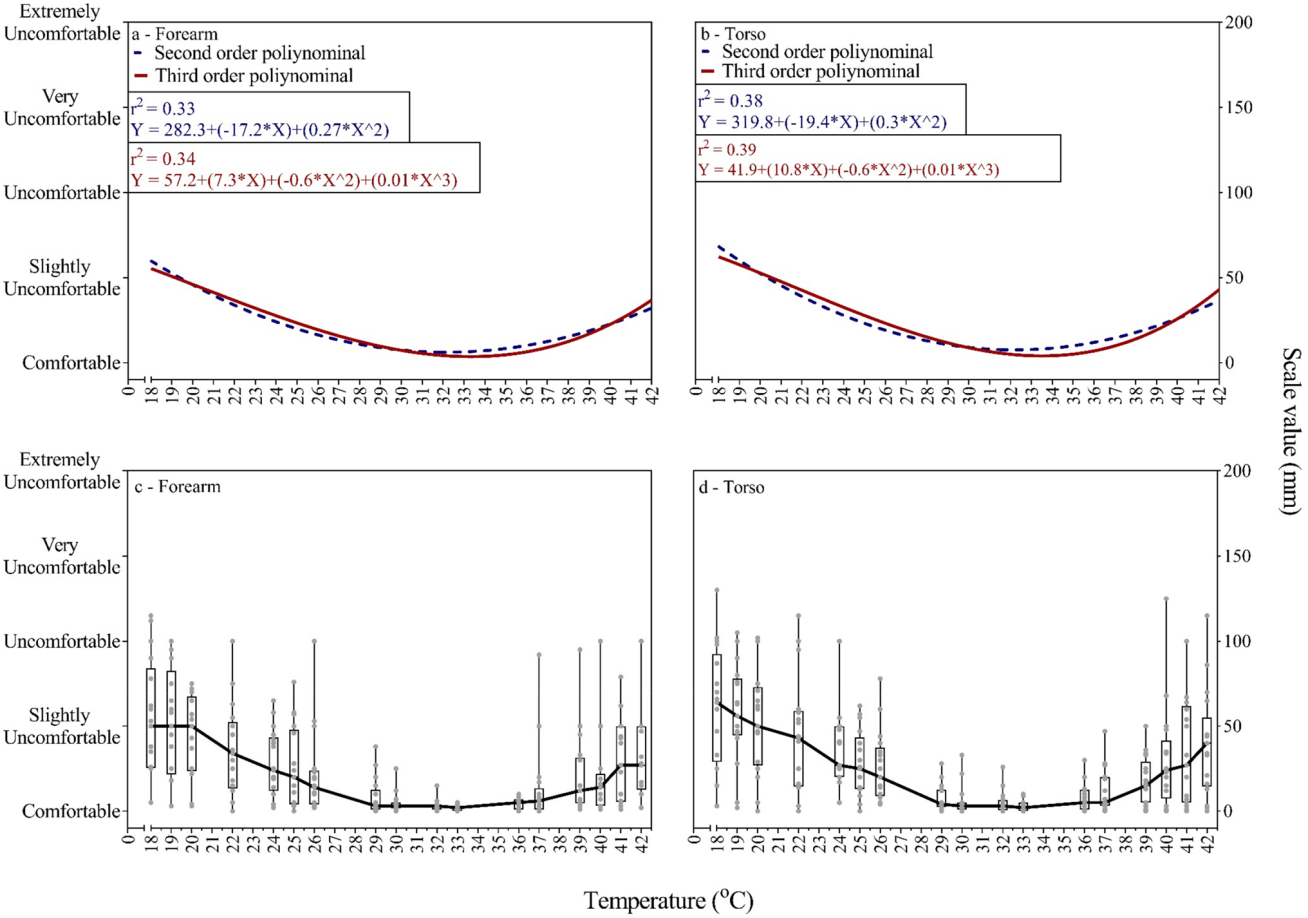
Relationship between applied physical temperature and thermal discomfort. Panel **a** and **b** demonstrate second (quadratic) (blue) and third (cubic) (blue) order polynomial regression lines of thermal discomfort in forearm (**a**) and torso (**b**). Panel **c** and **d** demonstrate individual data and box and whiskers with median connection line of thermal discomfort in forearm (**c**) and torso (**d**)

Second order (quadratic) and third order (cubic) regression analyses showed similar fits at both forearm (*r*^*2*^ = 0.33 and *r*^*2*^ = 0.34, respectively) and lateral torso (*r*^*2*^ = 0.38 and *r*^*2*^ = 0.39, respectively) test sites. Figure 3 (Panel a–b) shows the outcomes from the second order (quadratic) and third order (cubic) functions.

**Fig.4:**
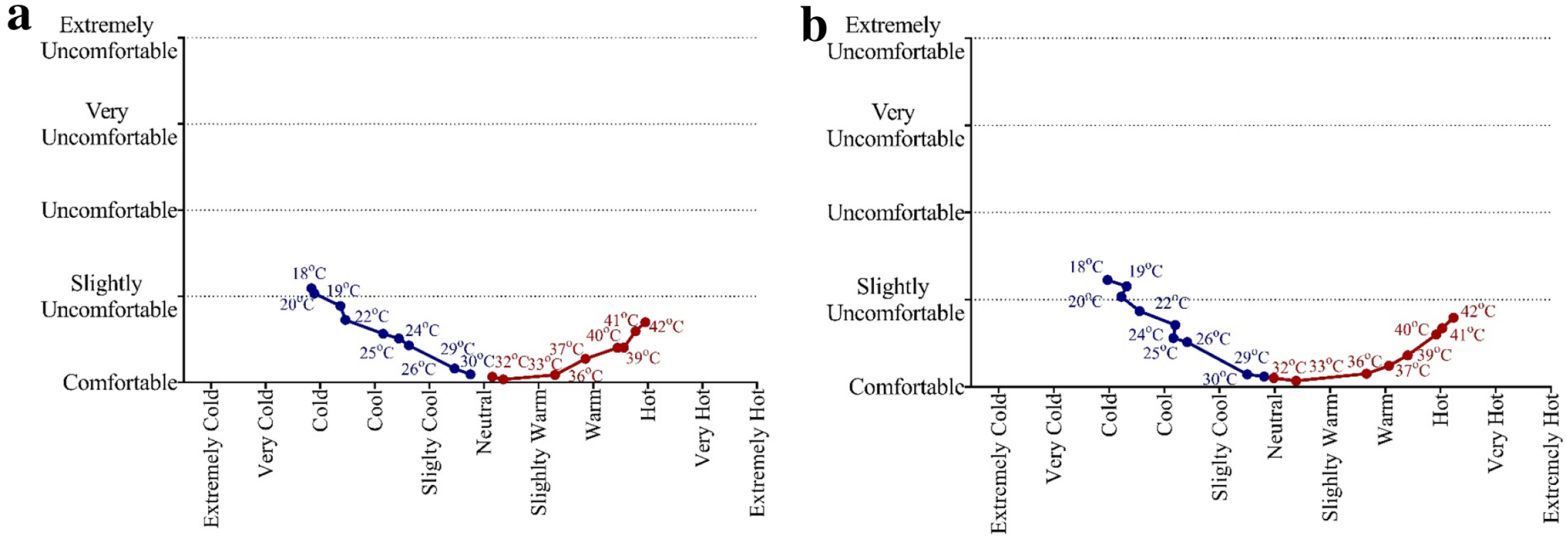
Correlation between thermal sensation and discomfort. Panel **a** demonstrates correlation between thermal sensation and discomfort in forearm. Panel **b** demonstrates correlation between thermal sensation and discomfort in torso

Participants perceived cold stimuli of 18 °C between “comfortable” and “uncomfortable”. When then stimulus temperature was 29–37 °C, they felt comfortable, and when it was further increased to 42 °C participants perceived it as between “comfortable” and “uncomfortable”. As seen in Fig. 4, the thermoneutral point was found between 31–32 °C for the local area. When a contact physical temperature was applied 10 degree above (42 °C) and 10 degree below (22 °C) this points, participants rated a similar level of thermal discomfort, as shown in Fig. 4. Thermal sensation and thermal comfort correlated linearly at 18–30 °C in both forearm and torso sessions (*r*^*2*^ = 0.97 and *r*^*2*^ = 0.94, respectively), and also at 32–42 °C across both body regions (*r*^*2*^ = 0.86 and *r*^*2*^ = 0.80, respectively), as shown in Fig. 4.

### Theoretical foundation 3: sex

There was no significant difference in thermal sensation or discomfort between male and female participants using a three-way mixed ANOVA analysis (*p* = 0.195 and* p* = 0.176, respectively). The variation between individuals in both forearm and torso sites is presented in Fig. 2 (Panels c–d). There was a wide-ranging variance in thermal discomfort perception across individuals, as shown in Fig. 3 (Panels c–d).

### Theoretical foundation 4: regional differences

The results of a three-way mixed ANOVA showed that the difference between thermal sensation in forearm and torso, also discomfort in forearm and torso sites, were not significantly different from one another (*p* = 0.297 and *p* = 0.179, respectively).

### Theoretical foundation 5: the confidence of thermal sensation

**Fig. 5:**
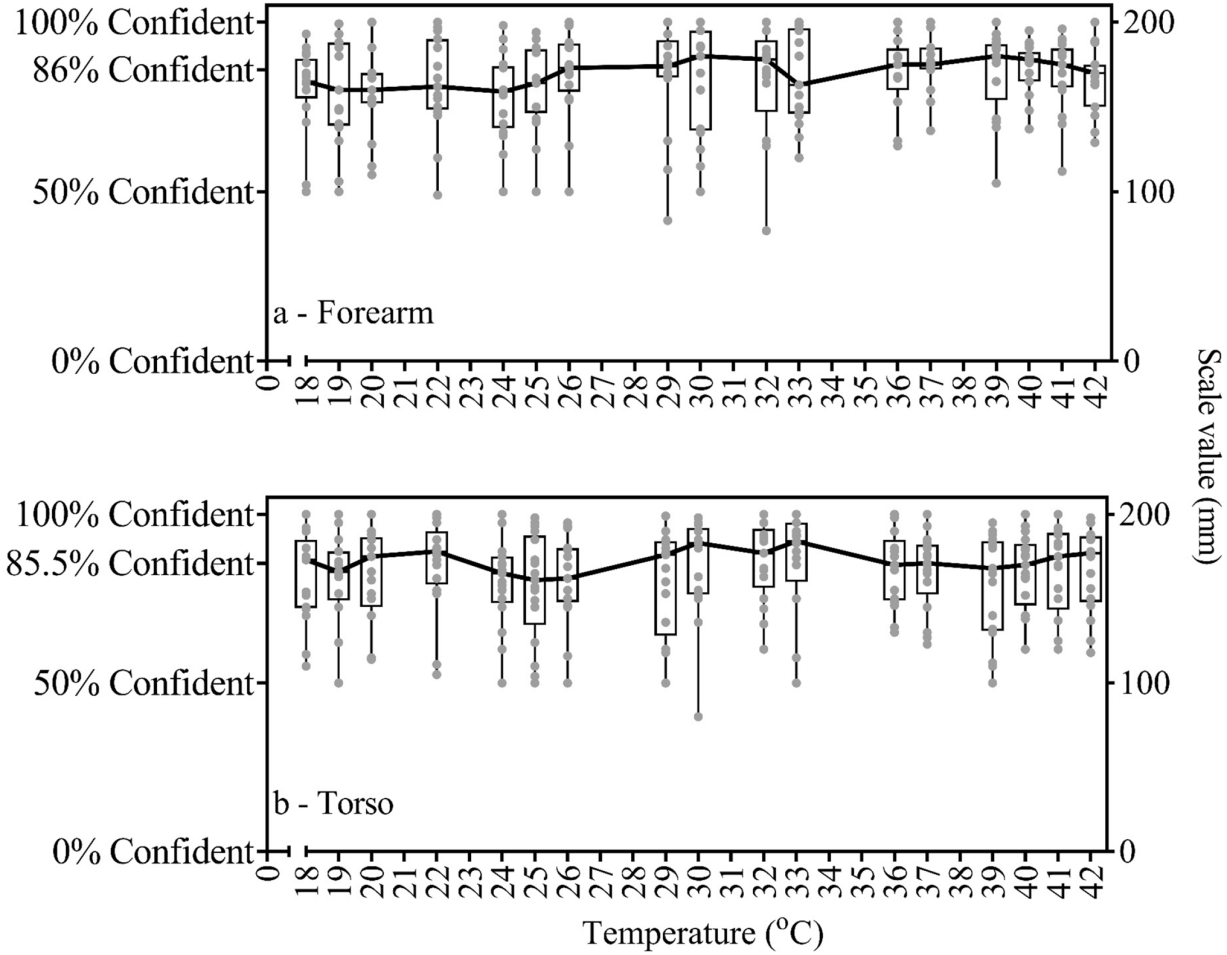
Individual data and box and whiskers with median connection line of the confidence of thermal sensation ratings in forearm (**a**), and in torso (**b**)

There was no correlation between the confidence in the perception of thermal sensation and the applied temperature across both body sites, for both male and female participants. Individuals’ confidence in their thermal sensation rating mean is 83.2% in forearm and 82.6% in the torso. The median scores are 86% for forearm and 85.5% for torso. The minimum and maximum confidence of thermal sensation rating is 38.5–100% in forearm and 40–100% in the torso, as illustrated in Fig. 5.

## Discussion

This study aimed to investigate the effects of physical contact temperatures on localized thermal sensation, thermal discomfort and on the confidence with which thermal sensations were experienced. Aligning with the aims of this study, five theoretical foundations are discussed.

### Theoretical foundation 1: thermal sensation relates linearly to physical contact temperature across a wide range

A limited number of research papers on thermal sensation have been published investigating the relationship between thermal sensation and temperature (Chatonnet and Cabanac 1965; Gagge et al. 1967; Zhang et al. 2004; Ouzzahra et al. 2012; Wang et al. 2015; Gerrett et al. 2015b). Hence this study characterized the relationship between thermal sensation and physical contact temperature, a factor that critically underpins our understanding of thermosensory testing. It was assumed that physical contact temperature and thermal sensation correlate linearly. Although the results of the experiment demonstrated that physical contact temperature in the range of 18–42 °C resulted in thermal sensation ratings that fit both positive linear and sigmoidal models (Fig. 2), the difference between the r-squared (*r*^*2*^) was small (1%). That may suggest that the distance between the thermal sensation anchors for the range studied is close to equal in terms of physical temperatures changes.

### Theoretical foundation 2: local thermal discomfort is more sensitive to cold than hot

The current study demonstrates a U-shaped (quadratic and cubic) relationship between the regional thermal discomfort and physical contact temperature, as expected. However, the second theoretical assumption tested in this study was that when a contact thermal probe was applied below the thermoneutral zone (i.e., cold), a participant would rate this as more uncomfortable than the same increase in contact temperature above the thermoneutral zone (i.e., hot). This assumption was based on previous research using whole-body thermal discomfort (Gagge et al. 1967). However, the present results showed that discomfort level was similar in both cold and hot for a given increase or decrease in physical contact temperature or thermal sensation, as shown in Fig. 4. One potential explanation for the contrary findings observed in whole-body research may be the development of separate evolutionary protective mechanisms for local and whole-body thermal stress. It could be speculated a local cold stimulus represents a lower threat to homeostasis compared to whole-body cooling, thereby not requiring the same degree of behavioral responses to prevent hypothermia. Moreover, humans typically overheat internally, so central thermoreceptors activation may lead to greater discomfort in the heat; whereas, humans typically cool from the outside in, so the activation of cutaneous thermoreceptors may lead to greater discomfort during to whole-body cold exposure (Gagge et al. 1967). It should also be considered that internal and skin temperature could change the relationship between physical contact temperatures and local thermal discomfort, and this should be the topic of future research (Cabanac 1969; Marks and Gonzalez 1974).

### Theoretical foundation 3: sex differences exist in thermal sensation and discomfort across a wide range of contact temperatures

It was assumed that there would be sex-related differences in the physical temperature to thermal sensation (Gerrett et al. 2014) and thermal discomfort relationships, across the a wide range of cold (18 °C) to hot (42 °C) thermal stimuli. The results from this investigation showed that there was no significant difference between male and female participants in their thermal sensation, confidence, and thermal discomfort ratings. Although these findings support to the results of the experiment conducted by Stevens and Choo (1996) showing that there was no sex-related differences in terms of threshold detection to warming and cooling, the findings are contrary to that of previous research using similar stimuli application methods to that of the present study (Gerrett et al. 2014). This apparent difference could however be explained by other methodological differences, such as normalization of sensation to skin temperature changes (sensitivity testing), use of single versus multiple contact temperature stimuli in the same experimental session, or analyzing a greater number of regions in comparison to previous studies.

### Theoretical foundation 4: regional thermal sensitivity differences exist independently of thermal discomfort changes, across a wide range of contact temperatures

In previous research it is implicitly assumed the regional differences in thermal sensation (Gerrett 2012; Gerrett et al. 2015b) are a direct result of spatial variations in the skin’s thermal sensitivity. However, it is possible that regional differences in thermal sensation can be explained, at least in part by a corresponding change in local thermal discomfort. The results suggest that there was no significant difference for participants` thermal sensation and thermal discomfort ratings between forearm and torso sites. This finding was contrary to that of previous research (Gerrett 2012; Gerrett et al. 2015b), with a possible explanation being that previous experiments applied thermal stimuli to various sites in the same session, allowing a more direct comparison between sites in that session. In contrast, in the current experiment, thermal stimuli were applied to different body sites across two separate sessions with same environmental conditions. This methodological difference, with concomitant difference in outcome, suggests that the temporal separation of the stimuli in the present experiment prevented participants directly comparing the two afferent thermo-sensory feedback signals from different body regions, thereby decreasing discrimination power between sites. To understand the key neuroscientific and psychophysiological mechanisms behind this finding, further investigations are crucial.

### Theoretical foundation 5: good levels of confidence exist in thermal sensation ratings across a wide range of contact temperatures

In the literature it is implicitly assumed that individual’s rate physical contact temperatures with a high level of certainty. However, it is also possible this may vary depending on the temperature and its proximity to the local thermoneutral zone, e.g., close to an extreme condition (cold and hot) participant rate with higher levels of confidence, than when rating near to the thermoneutral zone. The current results showed that the confidence of thermal sensation rating did not depend on physical contact temperature, and the median level of certainty was calculated at 86% for forearm and 85.5% for torso across the applied contact temperatures. A visual inspection of the confidence levels across individuals, indicates those participants with a lower confidence levels on average, also generally reported a higher variability in their confidence ratings, across the temperatures.

As understood from findings, participants scored lower confidence in their thermal sensation than expected, despite thermal sensation being a very basic and fundamental cutaneous sense, much like tactile, pain and itch sensing (Tominaga and Calerina 2004; McGlone and Reilly 2010; Goldstein 2013; Filingeri et al. 2015; Mather 2016). Given that thermal sensation is a basic and moderate fidelity-perception, it stands to reason that complex and multisensory perceptions such as wetness, stickiness, texture, comfort, fatigue or even ratings perceived exertion may be yield even lower levels of certainty than those observed for thermal sensations (Hollins et al. 1993; Bertaux et al. 2007; Filingeri et al. 2014a, 2015; Lloyd et al. 2016; Raccuglia et al. 2017; Maggie et al. 2018). This would have very important implications for our understanding of such complex perceptions and bring into question the validity of observations using sensorial scales for complex psychophysiological phenomena.

## Limitations and future research

A limitation of the present research is that the physical contact temperatures were selected such that the distribution was non-uniform (to limit the number of tests), while getting a good data representation across a wide span of temperatures. The present study was also limited to two testing body locations; thus, testing a greater number of locations may be beneficial for future research. Future research should also aim to better understand the role of sex, genotypic or phenotypic factors on local thermal sensation and/or discomfort perception level. To this end it would be beneficial to take a ‘matched-groups approach’ to examine sex differences, thereby nullifying the effect of e.g., body morphology in the present study. Indeed, it would be of interest to further investigate acclimation status, country of origin, and/or cultural factors, and how this may influence perception to local thermal stimuli (Chéry-Croze 1983; Schepers and Ringkamp 2010; Cândido et al. 2012; Mather 2016). Future research should also seek to investigate the thermal sensation relationship across a wider range, including the extremes of noxious hot and cold stimuli, using the same methods described herein. Lastly and perhaps most importantly, it will be of interest to determine how confidence in the ratings of complex perceptual phenomena, such as wetness perception, textile interactions and exercise-related sensations, vary across individuals.

## Conclusion

Thermal sensation is a complex phenomenon. The outlined study examined a range of theoretical foundations underpinning thermosensory testing, with key findings demonstrating that:

1. Physical contact temperatures in the range of 18–42 °C resulted in thermal sensation ratings that fit both positive linear and sigmoidal models. While the sigmoidal model provides an improvement in describing the data, the difference is very small, and thus, based on the linear model it may be concluded that the distance between the thermal sensation anchors for the range studied is close to equal.
2. Thermal discomfort evoked during the application of a conductive thermal probe follows a nonlinear U-shaped (quadratic and cubic) relationship with a physical applied temperature. When individuals experience a decrease in physical temperature below the thermoneutral zone, it is perceived as similarly uncomfortable as the same increases in physical temperature above the thermoneutral zone.
3. There were no sex-related differences in thermal sensation and discomfort across a wide range of physical contact temperatures.
4. The result of experiment conducted in two separate session showed that there was no significant difference for participants` thermal sensation and thermal discomfort ratings between forearm and torso sites across the wide range of physical temperature studied. When taken together with previous research, this suggests that participants are only able to discern regional differences in the same session, as they have a reference point for discrimination.
5. The median confidence of an individual’s thermal sensation rating was measured at 86%, ranging approximately 40–100%. This has important implications for research using subjective scales in the quantification of complex psychophysical stimuli, including wetness perception, textile interactions and exercise-related sensations.

The findings from this work are key in supporting the fundamental understanding and development of thermosensory testing and modelling.

## Abbreviations

ANOVA: Analysis of variance
BF: Body fat
RH: Relative humidity
SD: Standard deviation
